# Behavioral deficits in an Alzheimer’s Disease mouse model containing both APOE4 and Tau

**DOI:** 10.1002/alz.095029

**Published:** 2025-01-09

**Authors:** Aaron Booth, Samuel Neff, Vincenzo de Lillo, John Campitell, Amrah Marediya, Samantha Gray, Jane Flinn

**Affiliations:** ^1^ George Mason University, Vienna, VA USA

## Abstract

**Background:**

Alzheimer’s Disease (AD) is a neurological disease characterized by two major biological components; amyloid beta (abeta) and hyperphosphorylated tau. Research suggests that the hyperphosphorylated tau aggregation seen in late‐onset AD is characterized by two independent pathways, one caused by abeta buildup, and the other potentially caused by Apolipoprotein 4 (APOE4). However, research examining the relationship between hyperphosphorylated tau and APOE4 in the absence of abeta has been both inconsistent and lacks behavioral results. Likewise, research suggests that zinc exacerbates the effects of both tau and abeta in AD mouse models, however, there is limited research in how zinc interacts with APOE4.

**Method:**

We analyzed 8 groups of mice; 4 genotypes (WT, APOE4, Tau, APOE4xTau), 2 treatment groups (lixit water, zinc water), none of which contained abeta. Behavioral tests were run at 3 and 6 months of age to examine activities of daily living (Nesting & Burrowing), anxiety & motor ability (Open Field), and spatial memory (Morris Water Maze).

**Result:**

Initial results at 3 months indicate that Tau and APOE4xTau mice demonstrated impaired activities of daily living (Nesting; (p<.001), Burrowing (p<.001)), higher risk taking (p<.001), and impaired spatial memory (p<.001) compared to WT or APOE4 mice. APOE4 mice demonstrated improved spatial memory compared to WT; p<.001, and APOE4xTau mice demonstrated improved spatial memory compared to Tau; p<.001. However, APOE4 and APOE4xTau mice did not show differences in activities of daily living, nor anxiety compared to WT and Tau mice respectively. Additionally, Zinc did not significantly influence behavior at 3 months. Results will be re‐analyzed at 6 months of age, and brains will be analyzed for proteins of interest.

**Conclusion:**

Our initial findings provide evidence that APOE4 enhances certain behaviors at 3 months of age. This is consistent with previous research that APOE4 may show beneficial effects at a young age in both humans and mouse models. Subsequent analysis at 6 months will determine whether these beneficial effects continue at an older age.

